# Insufficient Lycopene Intake Is Associated With High Risk of Prostate Cancer: A Cross-Sectional Study From the National Health and Nutrition Examination Survey (2003–2010)

**DOI:** 10.3389/fpubh.2021.792572

**Published:** 2021-12-13

**Authors:** You Lu, Andrea Edwards, Zhong Chen, Tung-Sung Tseng, Mirandy Li, Gabrielle V. Gonzalez, Kun Zhang

**Affiliations:** ^1^Department of Physics and Computer Science, Xavier University of Louisiana, New Orleans, LA, United States; ^2^Behavioral and Community Health Science, School of Public Health, Louisiana State University Health Science Center, New Orleans, LA, United States; ^3^Bioinformatics Core of Xavier NIH RCMI Center of Cancer Research, Xavier University of Louisiana, New Orleans, LA, United States

**Keywords:** prostate cancer, PSA, lycopene, obesity, living status

## Abstract

Although lycopene intake and risk of prostate cancer have been explored for decades, recent studies show that Non-Hispanic Black Prostate Cancer (PCa) patients benefit less than Non-Hispanic White patients from a lycopene intake intervention program. This study examined whether a lycopene intake-related racial disparity exists in reducing the risk of PCa in healthy adults. Data on healthy, cancer-free Non-Hispanic Black (NHB) men (*n* = 159) and Non-Hispanic White (NHW) men (*n* = 478) from the 2003 to 2010 NHANES dataset were analyzed. Total lycopene intake from daily diet, age, living status, race/ethnicity, education level, poverty income ratio, body mass index, and smoking status were studied as independent variables. The combination of total Prostate-Specific Antigen (PSA) level and the ratio of free PSA was set as criteria for evaluating the risk of PCa. Multivariable logistic regression was used in race-stratified analyses to compute odds ratios (OR) and 95% confidence intervals (95% CI) comparing high PCa risk with low PCa risk. We found, in the whole population, race/ethnicity was the only factor that influenced lycopene intake from the daily diet. NHB men consumed less lycopene than NHW men (3,716 vs. 6,487 (mcg), *p* = 0.01). Sufficient lycopene intake could reduce the risk of PCa (OR: 0.40, 95% CI: 0.18–0.85, *p* = 0.02). Men aged between 66 and 70 had high PCa risk (OR: 3.32, 95% CI: 1.12–9.85, *p* = 0.03). Obesity served as a protective factor against the high risk of PCa (OR: 0.25, 95% CI: 0.12–0.54, *p* = 0.001). NHW men aged between 66 and 70 had a high risk of PCa (OR: 4.01, 95% CI: 1.02–15.73, *p* = 0.05). Obese NHW men also had lower risk of PCa (OR: 0.18, 95% CI: 0.07–0.47 *p* = 0.001). NHB men had a high risk of PCa compared to NHW men (OR: 2.27, 95% CI: 1.35–3.81 *p* = 0.004). NHB men who were living without partners experienced an even higher risk of PCa (OR: 3.35, 95% CI: 1.01–11.19 *p* = 0.07). Sufficient lycopene intake from daily food could serve as a protector against PCa. Such an association was only observed in NHW men. Further studies are needed to explore the dose-response relationship between lycopene intake and the association of PCa risk in NHB men.

## Introduction

The association between lycopene and the risk of prostate cancer (PCa) has been studied for over two decades (1). Lycopene can inhibit PCa cell growth and proliferation (2, 3), upregulate tumor suppressor genes (4), and lower oxidative DNA damage (5). Increased plasma lycopene levels can lower the risk of PCa (6, 7). However, lycopene absorption is highly associated with diet composition, in particular, the amount of fat (8) or calcium (9). Inconsistent lycopene exposure and PCa outcomes have been observed in Non-Hispanic Black (NHB) men and Non-Hispanic White (NHW) PCa patients (10). A recent study on dietary intake and TMPRSS2:ERG protein expression indicated that lycopene intake could lower the risk of death for ERG-fusion positive cases by 54%, but was not effective for ERG-fusion negative cases (11). ERG-fusion negative cases are mostly observed in NHB PCa patients compared to NHW PCa patients (12). Although aggressive PCa patients could benefit from taking an extra amount of lycopene (13), β-cryptoxanthin is more effective at protecting NHBs against aggressive PCa (14). Of note, for the healthy, metabolic syndrome-free population, no significant racial differences in lycopene intake have been observed (15). For this sake, it is necessary to explore the association between lycopene exposure and risk of PCa among NHB and NHW men.

Previous research suggests the lack of lycopene consumption in NHB men could be attributed to the individual level of knowledge, usage of supplemental diets, cultural values, and socioeconomic status. Black men commonly have a lower usage of supplemental diets than White men (16). This could be due to socioeconomic factors, such as lack of health insurance or regular visits with a primary care provider (16). Cultural practices and values dictate the dietary preferences of people from varying cultures and backgrounds. Since 2014, PCa trends have changed with an abundance of new cases and new deaths (16). This can all be taken into consideration when examining if a racial disparity exists in the intake of lycopene to reduce the risk of PCa developing in adult men.

The prostate-specific antigen (PSA) blood test is an effective but controversial method to diagnosis the risk of PCa. In general, 4.0 nanograms per milliliter (ng/mL) of blood is a well-accepted cutoff value for PCa risk. Recent evidence indicates free PSA level, prostate health index (17), PSA velocity (18), and 4Kscore (19) should also be considered in PCa screening for more accurate diagnosis. Age is one of the strongest risk factors that require extra attention when screening susceptible PCa risk populations. Men in their early 50's to later 60's could benefit from taking the PSA test (20). Men younger than 55 or older than 70 are not recommended to undergo a screening test unless it is proposed by the men themselves (21). NHB men or men with first-degree relatives who were diagnosed with PCa are a high-risk population, and therefore should consider taking the PSA test early (22). Other risk factors such as Body Mass Index (BMI) (23), smoking history (24), poverty income ratio (PIR) (25), education level (26), and marital status (27), should be taken into account as general confounders when measuring the risk of PCa for certain types of exposures.

The purpose of this study is to explore whether lycopene intake plays a different role in PCa prevention among different race groups. We constructed a multivariable logistic regression model using daily lycopene intake from the total diet as the exposure variable and the risk of PCa as the outcome. How numerical confounders, such as age, BMI, PIR, and descriptive variables, are associated with the risk of PCa were tested sequentially via stratification analysis after confirming that race acts as a moderator. To investigate why NHB individuals consume less lycopene than NHW individuals, subgroup comparisons were conducted based on preselected demographic and socioeconomic factors that were significantly associated with the risk of PCa.

## Methods

We extracted and combined four consecutive 2-year survey cycle datasets (2003–2004, 2005–2006, 2007–2008, 2009–2010) from the US Centers for Disease Control NHANES dataset for this study. A total of 637 male adults aged between 55 and 75 years old, with a completed response to the questions on lycopene intake and a recorded PSA level, were included (28). The lycopene consumption level from dietary was collected from two-day dietary interview questionnaires on total nutrient intakes, based on recall responses from survey participants. As a national survey, NHANES uses multistage, stratified, and probability clustering sampling methods under the supervision of the National Center for Health Statistics of the CDC (29). If participants met the selection criteria, an in-person face-to-face interview was conducted at the participant's home by trained staff. Individual demographic, health and nutrition data were collected through examination. In addition, collected blood and urine samples from the participants were sent to laboratories for further analysis (29). The National Center for Health Statistics Research Ethics Review Board revised and approved the survey protocol (30). Before starting the data collection process, a paper-based informed consent was signed by every participant. Our study was reviewed and approved by the Institutional Review Board of Xavier University of Louisiana.

## Measurements

The main exposure of interest for this study was lycopene intake. NHANES uses the five-step U.S. Department of Agriculture's Automated Multiple Pass Method (AMPM) to collect dietary intakes, including lycopene. More details on AMPM are provided in the NHANES dietary interviewer procedure manuals (31). A dose-response meta-analysis showed that 5 mg/day of lycopene intake could be the lowest amount that decreases the risk of PCa (32). We defined sufficient lycopene intake from daily food as ≥8000 mcg (33). Anyone who consumed zero lycopene or <8000 mcg was defined as lycopene intake insufficient. Smoking status was categorized into never smoker, former smoker, and current smoker based on the following questions: “Have you smoked at least 100 cigarettes in your entire life?” and “Do you now smoke cigarettes?” Never smoker was defined as a respondent who reported that they smoked <100 cigarettes in their lifetime; former smoker was defined as respondents who reported smoking ≥100 cigarettes in their lifetime and currently do not smoke cigarettes; current smoker was defined as respondents who reported smoking ≥100 cigarettes in their entire lifetime and were currently smoking every day or some days (34). The demographic information for included participants included age (55–59, 60–65, 6–70, 70–75), ace/ethnicity (Non-Hispanic White, Non-Hispanic Black), and education (less than high school and high school and above). Family PIR was defined by three consecutive levels (≤1.99, 2–2.99, and ≥3) after being adjusted for state-dependent gross income vs. the total capital per household across the nation. Living status was defined as living alone or with partners. Based on the martial status options, a participant who selected “married” or “living with a partner” was defined as living with a partner; a participant who selected “widowed,” “divorced,” “separated,” or “never married” were defined as living alone (35). In addition, BMI was classified as underweight/normal, overweight, and obese, respectively (36).

## Outcome

The risk of PCa in terms of PSA level was the primary outcome of interest for this study. We used a combination of total PSA and the ratio of free PSA to determine the risk of PCa. Specifically, high risk of PCa was defined as when total PSA ≥4.0 (ng/ml) and the ratio of free PSA was ≤25%; low risk of PCa was defined as when total PSA <4.0 (ng/ml) and the ratio of free PSA >25% (37). Subjects who were diagnosed with prostate infection, prostate cancer, or any type of cancer were excluded.

## Statistical Analysis

Participants' demographic, behavioral, and clinical features were summarized using descriptive statics, stratified by risk of PCa. The associations of these demographics with risk of PCa were validated using Rao-Scott Chi-square test for categorical variables and Fisher's exact test for small samples. A one-way ANOVA test was used to examine differences for continuous variables. Lycopene intake associated sampling weights were selected based on previous guidelines (38). All analyses were performed in R command (***svydesign***) by using the package “*survey*” (39) to create the weighted analysis groups for displaying percentage (%) (***svyciprop***) and means with standard error of the mean (***svymean***). Sampling weighted univariable logistic regression models (***svyglm***) were applied to examine the association between lycopene intake and high risk of PCa. We included the suggested confounders: age, race, living status, BMI, smoking status, PIR, and education level to construct the multivariable logistic regression model (24, 26, 27, 40, 41). A two-sample *t-*test (**svyttest**) was conducted for lycopene intake for different marital groups. All variables had no multicollinearity in the model. We followed the weighting instructions provided on the CDC website (42). All analyses were conducted using R version 3.6.3 with packages “*survey*” and “*dplyr*.” All tests were 2-sided, and a *P* < 0.05 was considered statistically significant for all tests.

## Result

A total of 637 cancer-free male adults aged between 55 and 75 years were included from NHANES 2003–2010. Table 1 provides weighted percentage and raw sample sizes for demographics and lycopene intake by risk of PCa—high PCa risk (total PSA ≥4.0 (ng/ml) and ratio of free PSA ≤25%) and low PCa risk (total PSA <4.0 ng/ml) and ratio of free PSA >25%). For the overall study population, 28.4% of participants (*n* = 155) had sufficient lycopene intake; 71.6% of participants (*n* = 482) had insufficient lycopene intake. People in the low PCa risk group consumed more lycopene than people in the high PCa risk groups: 6471 ± 456, mcg vs. 4287 ± 461, mcg, *p* = 0.003. The average age in the low PCa risk group was younger than the high PCa risk group: 62.7 ± 0.3 vs. 65.2 ± 0.9, *p* = 0.01. The total PSA level and free PSA ratios were 1.03 ± 0.04 ng/ml, 37.93 ± 0.61% in the low PCa risk group and, 6.52 ± 0.34 ng/ml, 16.87 ± 0.64% in the high PCa risk group, respectively. Although the living condition was not distributed differently among the two risk groups (*p* = 0.42), the living alone group had more people with high PCa risk than the living with partners group: 11.5 vs. 8.9%. Age has been strongly associated with the risk of PCa. The percentage of high PCa risk increased from the middle 50's group to the middle 70's group: 5.0–13.3%, *p* = 0.04. The NHB population had more people with the high PCa risk than the NHW population: 18.0 vs. 8.7 %, *p* = 0.0006. Families with high incomes, PIR ≥ 3, had low PCa risk compared to those who were lower-income, PIR <1.99: 8.3 vs. 10.0%, *p* = 0.34, respectively. Interestingly, the obese population had a lower percentage of high-risk PCa individuals than the under/normal weight population, 5.1 vs. 17.3%, respectively, *p* = 0.002. Overall, lycopene intake, age, race, and BMI were risk factors significantly associated with risk of PCa.

**Table 1:** Characteristics of 637 male adults with different PCa risk using NHANES 2003–2010 Data.

**Characteristic**	**Overall**	**Low PCa Risk**	**High PCa Risk**	***P*** **-value**
**Total**, ***n*** **(%)**	637	557 (90.6)	80 (9.4)	
Mean total PSA ± SEM (ng/mL)	1.55 ± 0.08	1.03 ± 0.04	6.52 ± 0.34	<0.00001^a^
Mean free PSA ratio ± SEM (%)	35.94 ± 0.65	37.93 ± 0.61	16.87 ± 0.64	<0.00001^a^
Mean lycopene Intake ± SEM (mcg)	6,265 ± 428	6,471 ± 456	4,287 ± 461	0.003^a^
Mean age ± SEM (year)	62.9 ± 0.3	62.7 ± 0.3	65.2 ± 0.9	0.01^a^
**Lycopene intake**, ***n*** **(%)**				0.03^b^
Insufficient	482 (71.6)	418(88.8)	64 (11.2)	
Sufficient	155 (28.4)	139 (95.1)	16 (4.9)	
**Living status**, ***n*** **(%)**				0.42^c^
With partners	484 (80.3)	429 (91.1)	55 (8.9)	
Alone	153 (19.7)	128 (88.5)	25 (11.5)	
Age, *n* (%)				0.04^b^
55–59	141(35.0)	130 (95.0)	11(5.0)	
60–65	174 (27.0)	157 (90.5)	17 (9.5)	
66–70	178 (22.7)	150 (86.3)	28 (12.7)	
71–75	144 (15.4)	120 (86.7)	24 (13.3)	
**Race**, ***n*** **(%)**				0.0006^c^
Non-Hispanic White	478 (92.0)	428 (91.3)	50 (8.7)	
Non-Hispanic Black	159 (8.0)	129(82.0)	30 (18.0)	
**Education level**, ***n*** **(%)**				0.98^c^
Less than high school	153 (15.9)	132 (90.6)	21 (9.4)	
High school and above	484 (84.1)	425 (90.5)	59 (9.5)	
**PIR**, ***n*** **(%)**				0.34^b^
1.99	225 (22.0)	193 (90.0)	32 (10.0)	
2–2.99	102 (16.8)	88 (87.0)	14 (13.0)	
3	310 (61.3)	276 (91.7)	34 (8.3)	
**BMI**, ***n*** **(%)**				0.002^c^
Under/normal weight	133 (18.8)	106 (82.7)	27 (17.3)	
Overweight	278 (46.0)	247 (90.4)	31 (9.6)	
Obese	226 (35.2)	204 (94.9)	22 (5.1)	
**Smoking Status**, ***n*** **(%)**				0.66^b^
Non-smoker	218 (35.1)	188 (89.3)	30 (10.7)	
Ever smoker	300 (49.7)	268 (90.9)	32 (9.1)	
Current smoker	119 (15.2)	101 (92.2)	18 (7.8)	

*One-way ANOVA^a^ test found total PSA, free PSA ratio, lycopene intake, and age are distributed differently between low and high PCa risk groups.*

*The categorical analysis found race/BMI (Rao-Scott Chi-square^c^ test)*,

*lycopene intake/age (Fisher's exact^b^ test) have statistically different distribution between low and high PCa risk groups. SEM, standard error of the mean; NHANES, National Health and Nutrition Examination Survey; PCa, prostate cancer; BMI, body mass index; PIR, Poverty Income Ratio*.

The computed results from weighted logistic regression models are given in Table 2. In raw analyses, individuals with sufficient lycopene intake had 0.41 times lower odds (95% CI: 0.21–0.78) of having a high risk of PCa. However, NHB individuals had 2.29 times greater odds (95% CI: 1.40–3.75) of having a high risk of PCa. Of note, obese people only had 0.25 times lower odds (95% CI: 0.11–0.52) of having a high risk of PCa. Further moderation analysis confirmed race as a moderator between lycopene intake associated high risk of PCa (*p* = 0.05). After adjusting for confounders, lycopene intake, race, and BMI were still statistically significantly associated with a high risk of PCa (ORs: 0.40, 2.27, 0.25, 95% CIs: 0.18–0.85, 1.35–3.81, and 0.12–0.54, respectively). People between ages 66 and 70 had a significantly higher risk of PCa (OR: 3.32, 95% CI: 1.12–9.85).

**Table 2:** Factors associated with a high risk of PCa (Total PSA 4.0 ng/ml and ratio of free PSA 25%).

**Characteristic**	**Unadjusted OR**	**95% CI**	***P*** **-value**	**Adjusted OR**	**95% CI**	***P*** **-value**
**Lycopene intake**						
Insufficient (ref)	1			1		
Sufficient	0.41	0.21–0.78	0.01	0.4	0.18–0.85	0.02
**Living status**						
With partners (ref)	1			1		
Alone	1.32	0.66–2.65	0.43	1.29	0.58–2.86	0.53
**Age**						
55–59 (ref)	1			1		
60–65	1.99	0.63–6.25	0.24	2.28	0.76–6.82	0.15
66–70	3.02	0.89–10.18	0.08	3.32	1.12–9.85	0.03
71–75	2.91	0.98–8.62	0.06	2.8	1.00–7.85	0.05
**Race**						
Non-Hispanic White (ref)	1			1		
Non-Hispanic Black	2.29	1.40–3.75	0.0001	2.27	1.35–3.81	0.004
**Education level**						
Less than high school (ref)	1			1		
High school and above	1.01	0.48–2.10	0.98	1.14	0.53–2.43	0.73
**PIR**						
1.99 (ref)	1			1		
2–2.99	1.35	0.57–3.15	0.49	1.39	0.59–3.28	0.31
3	0.81	0.45–1.44	0.48	1.16	0.55–2.43	0.58
**BMI**						
Under/normal weight (ref)	1			1		
Overweight	0.5	0.25–1.01	0.06	0.55	0.28–1.06	0.08
Obese	0.25	0.11–0.52	0.0005	0.25	0.12–0.54	0.001
**Smoking status**						
Non-smoker (ref)	1			1		
Ever smoker	0.83	0.40–1.69	0.61	0.84	0.41–1.71	0.64
Current smoker	0.7	0.33–1.51	0.37	0.6	0.27–1.36	0.23

*The adjusted linear logistic regression models show that a high risk of PCa is associated with lycopene intake (P = 0.02), race (P = 0.004), and obesity (P = 0.001). The interaction term (race and lycopene intake) had a statistical significance (P = 0.04). OR, odds ratio; CI, confidence interval; BMI, body mass index; PCa, prostate cancer; PIR, Poverty Income Ratio*.

Factors that are associated with a high risk of PCa across race groups are listed in Table 3. For NHW individuals, the sufficient lycopene intake population had 0.31 times lower odds (95% CI: 0.12–0.81, *p* = 0.02) of having a high risk of PCa. NHB individuals who were living alone had 3.35 times greater odds (95% CI: 1.01–11.19, *p* = 0.07) of having a high risk of PCa. For ages 66–70, NHW individuals had 4.01 times greater odds (95% CI: 1.02–15.73, *p* = 0.05) of having high risk of PCa. In addition, obese NHW individuals had 0.18 times lower odds (95% CI: 0.07–0.47, *p* = 0.001) of having a high risk of PCa. An association of high PCa risk and other confounders (i.e., PIR, smoking, and education level) was not observed. Overall, mean lycopene intake was significantly lower in the NHB group (3,716 ± 591 mcg) than the NHW group (6,487 ± 452 mcg). NHB individuals averaged 1,851 ± 550 mcg lycopene when compared to NHW individuals, who averaged 6,062 ± 1,247 mcg lycopene per day if they were living alone. For those ages 66–70, NHB individuals consumed less lycopene than NHW individuals (p = 0.02). In addition, overweight NHW individuals consumed more lycopene (6,663 ± 815 mcg) than overweight NHB individuals (3,197 ± 648 mcg). More comparison details on living status, age, and BMI on lycopene intake across different race groups are given in Table 4.

**Table 3:** Factors associated with a high risk of PCa across different race groups.

**Characteristics**	**Non-Hispanic Black**			**Non-Hispanic White**		
	**Adjusted OR**	**95% CI**	***P*** **-value**	**Adjusted OR**	**95% CI**	***P*** **-value**
**Lycopene**						
Insufficient (ref)	1			1		
Sufficient	2.67	0.81–8.83	0.13	0.31	0.12–0.81	0.02
**Living status**						
With partners (ref)	1			1		
Alone	3.35	1.01–11.19	0.07	1.17	0.43–3.18	0.75
**Age**						
55–59 (ref)	1			1		
60–65	0.39	0.08–1.82	0.26	3.13	0.78–12.55	0.12
66–70	1.48	0.39–5.55	0.57	4.01	1.02–15.73	0.05
71–75	3.85	0.74–19.98	0.13	3.03	0.81–11.35	0.11
**BMI**						
Under/normal weight (ref)	1			1		
Overweight	0.36	0.09–1.41	0.17	0.53	0.26–1.06	0.08
Obese	0.79	0.22–2.81	0.73	0.18	0.07–0.47	0.001

*All variables had no multicollinearity in the adjusted model. The adjusted linear logistic regression models found a high risk of PCa is associated with living status in the Non-Hispanic Black population (P = 0.07). For Non-Hispanic White, insufficient lycopene intake (P = 0.02) and obesity (P = 0.001) are associated with a high risk of PCa. CI, confidence interval; PIR, poverty income ratio; BMI, body mass index; OR, odds ratio*.

**Table 4:** Non-Hispanic Black individuals consume less lycopene than Non-Hispanic White individuals.

**Characteristics**	**Non-Hispanic Black**	**Non-Hispanic White**	***P*** **-value**
Sufficient, *n* (%)	26 (16.8)	129 (29.4)	
Insufficient, *n* (%)	133 (83.2)	349 (70.6)	
**Mean lycopene intake** **±** **SEM, (%)**			
Overall	3,716 ± 591	6,487 ± 452	0.01^a^
Living status	0.01^b^	0.7^b^	
With partners	4,304 ± 741, (76.0)	6,859 ± 498, (80.6)	0.13^a^
Alone	1,851 ± 550, (24.0)	6,062 ± 1247, (19.4)	0.02^a^
**Age (%)**			
55–59	5,172 ± 1,181, (34.6)	6,351 ± 1,027, (35.0)	0.73^a^
60–65	3,955 ± 813, (29.0)	6,393 ± 712, (26.8)	0.05^a^
66–70	2,087 ± 758, (24.1)	6,772 ± 830, (22.5)	0.02^a^
71–75	2,243 ± 958, (12.3)	6,612 ± 1,097, (15.7)	0.23^a^
**BMI (%)**			
Under/normal weight	2,801 ± 775, (28.1)	5,821 ± 953, (18.0)	0.16^a^
Overweight	3,197 ± 648, (38.0)	6,663 ± 815, (46.7)	0.04^a^
Obese	5,054 ± 1,416, (33.9)	6593 ± 589, (35.3)	0.34^a^

*Non-Hispanic Black when living alone, or at the age between 66 and 70, or BMI following the overweight range have significantly lower lycopene intake than Non-Hispanic White*.

*Statistical test using one-way ANOVA^a^*

*and two-sample t-test^b^*.

*SD, standard deviation; BMI, body mass index; SEM, standard error of the mean*.

## Discussion

This study investigated the association between lycopene intake from daily food and the risk of PCa by using accumulated NHANES datasets. Major findings of this study indicate that sufficient lycopene intake could reduce the risk of PCa. This association, however, was observed in NHW individuals only. This disparity could be due to several factors. Consistent with a previous study reporting that NHB individuals usually consume less lycopene (28), we found that only 16.8% of NHB respondents had sufficient intake, compared to 29.4% of NHW respondents. Stratification analysis using pre-determined independent PCa risk factors confirmed living alone is a major barrier for NHB individuals to consume lycopene. Future work should focus on the interaction between living status and lycopene intake for the prevention of the PCa risk in NHB individuals.

NHB individuals consume less lycopene from daily food than NHW individuals (40). Of note, NHB individuals with sufficient lycopene intake had an associated high risk of PCa (OR: 2.67, 95% CI: 0.81–8.83, *p* = 0.13). For the high PCa risk group, overweight or obese NHB individuals consumed more lycopene, but this was not the case for NHW individuals. Furthermore, more overweight and obese NHB individuals had a high PCa risk than NHW individuals (Table 5). Such inconsistent observations might explain why a reverse association occurs among NHB individuals. In addition, age is a well-known risk factor of PCa, although lycopene intake was slightly higher in NHW individuals aged 66–70 than aged 55–59, (6,772 ± 830 mcg vs. 6,351 ± 1,027 mcg), this trend was not found to be statistically significant after conducting a two-sample *t*-test (*p* = 0.78).

**Table 5:** Comparison of lycopene intake (mcg) between PCa risk groups under different BMI conditions.

**Characteristics**	**Mean lycopene intake** **±** **SEM, (%)**			
	**Non-Hispanic Black**		**Non-Hispanic White**	
BMI	Low PCa Risk	High PCa Risk	Low PCa Risk	High PCa Risk
Under/normal weight	3,353 ± 967, (76.9)	958 ± 442, (23.1)	6,227 ± 1,115, (83.5)	3,767 ± 1,090, (16.5)
Overweight	3,110 ± 702, (88.0)	3,833 ± 1,392, (12.0)	6,944 ± 920, (90.6)	3,963 ± 598, (9.4)
Obese	3,506 ± 1,118, (79.6)	11,088 ± 3,983, (20.4)	6,656 ± 604, (96.2)	4,989 ± 1,162, (3.8)

*PCa, prostate cancer; BMI, body mass index; SEM, standard error of the mean*.

The association between the risk of PCa and lycopene intake was derived based on the filtering standard for PCa risk. Although a lower cut-off value for total PSA (i.e., 2 ng/ml) could better predict PCa risk in a four-year surveillance interval (43), we decided to use the most widely accepted cut-off value (4 ng/ml) for total PSA, then combined the ratio of free PSA to address the heterogeneity of PSA measurement across NHB and NHW individuals (37). Based on re-defined filtering criteria, we confirmed that age is the main contributor to the high risk of PCa (44). The average age of the sampled population was 62.9 years old, and age was distributed differently across the two PCa risk groups. NHW individuals aged between 66 and 70 had a higher risk of PCa. Overall, sufficient lycopene intake could reduce this high risk of PCa. Such a conclusion is consistent with findings from other studies (45, 46). Further interaction analysis confirmed the role of race/ethnicity as a moderator between lycopene intake and the risk of PCa. Except for lycopene intake, other co-variables that were associated with a high risk of PCa did not share the same patterns between NHB and NHW individuals. Consistent with a previous study that found that obesity is associated with higher plasma lycopene levels (47), our multivariable logistic regression model confirmed that obesity is negatively associated with a higher risk of PCa in the NHW population (41).

This study has some limitations. First, NHANES relies on self-reported data, including data on height and weight that are required for computing BMI. Second, only 2003–2010 data were used to derive the association between lycopene intake and risk of PCa. This is because this period was the maximum time length that allowed us to use matched PSA data based on our selection criteria. Finally, to explore the association between exposures and outcome, the coefficients were calculated sequentially by regular multiple regression models. The actual contribution of sufficient lycopene intake on reducing the risk of PCa may be affected by other nutrient consumption. More advanced analysis methods are required to address this issue in further studies (48).

## Conclusion

A racial disparity in lycopene intake associated with the risk of PCa was observed in this study. Sufficient lycopene intake could be protective against the high risk of PCa, but such an effect was observed in the NHW population only. Overall, NHB individuals consumed less lycopene from their daily diet than NHW individuals. Age, BMI, and living status affected lycopene intake differently between NHB and NHW individuals. Extra attention should be given to these variables when designing lycopene-based nutrition programs for PCa prevention in the NHB population.

## Data Availability Statement

Publicly available datasets were analyzed in this study. This data can be found at: https://www.cdc.gov/nchs/nhanes/index.htm.

## Ethics Statement

The studies involving human participants were reviewed and approved by the study used public available datasets that meet the Federal Regulation at 46 CFR 46.102. The patients/participants provided their written informed consent to participate in this study.

## Author Contributions

YL contributed to the conception, design, and database organization of the study. YL and ZC performed the statistical analysis. AE, ML, and GG cross-validated this study. T-ST and KZ supervised this study. All authors contributed to manuscript writing.

## Funding

This work was partly supported by funding from the National Institutes of Health (NIH) grant U54MD007595.

## Author Disclaimer

The contents are solely the responsibility of the authors and do not necessarily represent the official views of the NIH.

## Conflict of Interest

The authors declare that the research was conducted in the absence of any commercial or financial relationships that could be construed as a potential conflict of interest.

## Publisher's Note

All claims expressed in this article are solely those of the authors and do not necessarily represent those of their affiliated organizations, or those of the publisher, the editors and the reviewers. Any product that may be evaluated in this article, or claim that may be made by its manufacturer, is not guaranteed or endorsed by the publisher.

## References

[1] Clinton SK Giovannucci E. Diet, nutrition, and prostate cancer. Annu Rev Nutr. (1998) 18:413–40. 10.1146/annurev.nutr.18.1.413 9706231

[2] Ford NA Elsen AC Zuniga K Lindshield BL Erdman JW. Lycopene and apo-12′-lycopenal reduce cell proliferation and alter cell cycle progression in human prostate cancer cells. Nutr Cancer. (2011) 63:256–63. 10.1080/01635581.2011.523494 21207319

[3] Mirahmadi M Azimi-Hashemi S Saburi E Kamali H Pishbin M Hadizadeh F. Potential inhibitory effect of lycopene on prostate cancer. Biomed Pharmacother. (2020) 129:110459. 10.1016/j.biopha.2020.110459 32768949

[4] Wertz K. Lycopene effects contributing to prostate health. Nutr Cancer. (2009) 61:775–83. 10.1080/01635580903285023 20155615

[5] Chen L Stacewicz-Sapuntzakis M Duncan C Sharifi R Ghosh L Breemen RV. Oxidative DNA damage in prostate cancer patients consuming tomato sauce-based entrees as a whole-food intervention. J Natl Cancer Inst. (2001) 93:1872–9. 10.1093/jnci/93.24.1872 11752012

[6] Dahan K Fennal M Kumar NB. Lycopene in the prevention of prostate cancer. J Soc Integr Oncol. (2008) 6:29–36. 10.2310/7200.2008.0001 18302908

[7] Gann PH Ma J Giovannucci E Willett W Sacks FM Hennekens CH. Lower prostate cancer risk in men with elevated plasma lycopene levels: results of a prospective analysis. Cancer Res. (1999) 59:1225–30. 10096552

[8] Moran NE Erdman JW Jr Clinton SK. Complex interactions between dietary and genetic factors impact lycopene metabolism and distribution. Arch Biochem Biophys. (2013) 539:171–80. 10.1016/j.abb.2013.06.017 23845854 PMC3818361

[9] Borel P Desmarchelier C Dumont U Halimi C Lairon D Page D. Dietary calcium impairs tomato lycopene bioavailability in healthy humans. Br J Nutr. (2016) 116:2091–6. 10.1017/S0007114516004335 28069089

[10] Vogt TM Mayne ST Graubard BI Swanson CA Sowell AL Schoenberg JB. Serum lycopene, other serum carotenoids, and risk of prostate cancer in US blacks and whites. Am J Epidemiol. (2002) 155:1023–32. 10.1093/aje/155.11.1023 12034581

[11] Graff RE Pettersson A Lis RT DuPre N Jordahl KM Nuttall E. The TMPRSS2:ERG fusion and response to androgen deprivation therapy for prostate cancer. Prostate. (2015). 75:897–906. 10.1002/pros.22973 25728532 PMC4424159

[12] Rosen P Pfister D Young D Petrovics G Chen Y Cullen J. Differences in frequency of ERG oncoprotein expression between index tumors of Caucasian and African American patients with prostate cancer. Urology. (2012). 80:749–53. 10.1016/j.urology.2012.07.001 22950997 PMC3462242

[13] Wertz K Siler U Goralczyk R. Lycopene: modes of action to promote prostate health. Arch Biochem Biophys. (2004) 430:127–34. 10.1016/j.abb.2004.04.023 15325920

[14] Antwi SO Steck SE Su LJ Hebert JR Zhang H Craft NE. Carotenoid intake and adipose tissue carotenoid levels in relation to prostate cancer aggressiveness among African-American and European-American men in the North Carolina–Louisiana prostate cancer project (PCaP). Prostate. (2016). 76:1053–66. 10.1002/pros.23189 27271547 PMC5080909

[15] Paydar M Johnson AA. Dietary intake, physical activity and metabolic syndrome in African Americans, hispanics and whites. J Natl Med Assoc. (2020) 112:215–24. 10.1016/j.jnma.2019.11.005 32067763

[16] Bhardwaj A Srivastava SK Khan MA Prajapati VK Singh S Carter JE. Racial disparities in prostate cancer: a molecular perspective. Front Biosci (Landmark Ed). (2017) 22:772–82. 10.2741/4515 27814645 PMC5242333

[17] Jansen FH van Schaik RH Kurstjens J Horninger W Klocker H Bektic J. Prostate-specific antigen (PSA) isoform p2PSA in combination with total PSA and free PSA improves diagnostic accuracy in prostate cancer detection. Eur Urol. (2010) 57:921–7. 10.1016/j.eururo.2010.02.003 20189711

[18] Vickers AJ Thompson IM Klein E Carrol PR Scardino PT. A commentary on PSA velocity and doubling time for clinical decisions in prostate cancer. Urology. (2014) 83:592–8. 10.1016/j.urology.2013.09.075 24581521

[19] Loeb S Lilja H Vickers A. Beyond PSA: utilizing novel strategies to screen men for prostate cancer. Curr Opin Urol. (2016) 26:459–65. 10.1097/MOU.0000000000000316 27262138 PMC5035435

[20] Carter HB Albertsen PC Barry MJ Etzioni R Freedland SJ Greene KL. Early detection of prostate cancer: AUA guideline. J Urol. (2013) 190:419–26. 10.1016/j.juro.2013.04.119 23659877 PMC4020420

[21] Bell N Connor Gorber S Shane A Joffres M Singh H Dickinson J. Recommendations on screening for prostate cancer with the prostate-specific antigen test. CMAJ. (2014) 186:1225–34. 10.1503/cmaj.140703 25349003 PMC4216256

[22] Smith RA Cokkinides V von Eschenbach AC Levin B Cohen C Runowicz CD. American Cancer Society guidelines for the early detection of cancer. CA Cancer J Clin. (2002) 52:8–22. 10.3322/canjclin.52.1.8 11814067

[23] Wright JL Lin DW Stanford JL. The effect of demographic and clinical factors on the relationship between BMI and PSA levels. Prostate. (2011) 71:1631–7. 10.1002/pros.21380 21432865 PMC3409087

[24] De Nunzio C Andriole GL Thompson IM Jr Freedland SJ. Smoking and prostate cancer: a systematic review. Eur Urol Focus. (2015) 1:28–38. 10.1016/j.euf.2014.10.002 28723351

[25] Kao YH LinWT Thomas CL Lin HY Tseng TS. Association between smoking and neutrophil to lymphocyte ratio among prostate q18 cancer survivors: the 2005-2016 national health and nutrition examination survey. In: APHA's (2019). Annual Meeting and Expo (Nov. 2-Nov. 6) American Public Health Association. Philadelphia, PA (2019).

[26] Winterich JA Grzywacz JG Quandt SA Clark PE Miller DP Acuña J. Men's knowledge and beliefs about prostate cancer: education, race, and screening status. Ethn Dis. (2009) 19:199–203. 10.18865/ed.31.4.527 19537233 PMC2699598

[27] Tyson MD Andrews PE Etzioni DA Ferrigni RG Humphreys MR Swanson SK. Marital status and prostate cancer outcomes. Can J Urol. (2013) 20:6702–6. 10.1016/j.juro.2012.02.222 23587510

[28] Kirsh VA Mayne ST Peters U Chatterjee N Leitzmann MF Dixon LB. A prospective study of lycopene and tomato product intake and risk of prostate cancer. Cancer Epidemiol Biomarkers Prev. (2006) 15:92–8. 10.1158/1055-9965.EPI-05-0563 16434593

[29] Centers for Disease Control Prevention. National Health and Nutrition Examination Survey. Available online at: http://www.cdc.gov/nchs/nhanes.htm (accessed August 19, 2021).

[30] Centers for Disease Control Prevention. National Health and Nutrition Examination Survey. NCHS Research Ethics Review Board (ERB) Approval. Available online at: https://www.cdc.gov/nchs/nhanes/irba98.htm (accessed August 19, 2021).

[31] Centers for Disease Control Prevention. National Health and Nutrition Examination Survey. Available online at: https://www.cdc.gov/nchs/data/nhanes/nhanes_03_04/DIETARY_MEC.pdf (accessed August 19, 2021).

[32] Chen P Zhang W Wang X Zhao K Negi DS Zhou L. Lycopene and risk of prostate cancer: a systematic review and meta-analysis. Medicine. (2015) 94:e1260. 10.1097/MD.0000000000001260 26287411 PMC4616444

[33] Giovannucci E Rimm EB Liu Y Stampfer MJ Willett WC. A prospective study of tomato products, lycopene, and prostate cancer risk. J Natl Cancer Inst. (2002) 94:391–8. 10.1093/jnci/94.5.391 11880478

[34] McClave AK Dube SR Strine TW Mokdad AH. Associations between health-related quality of life and smoking status among a large sample of US adults. Prev Med. (2009) 48:173–9. 10.1016/j.ypmed.2008.11.012 19103219

[35] Sanderson M Coker AL Perez A Du XL Peltz G Fadden MK. A multilevel analysis of socioeconomic status and prostate cancer risk. Ann Epidemiol. (2006) 16:901–7. 10.1016/j.annepidem.2006.02.006 16843007 PMC5508518

[36] Center, for Disease Control Prevention. Continuous NHANES. Available online at: https://wwwn.cdc.gov/nchs/nhanes/continuousnhanes/default.aspx (accessed August 19, 2021).

[37] Flynn-Evans EE Mucci L Stevens RG Lockley SW. Shiftwork and prostate-specific antigen in the National Health and Nutrition Examination Survey. J Natl Cancer Inst. (2013). 105:1292–7. 10.1093/jnci/djt169 23943864 PMC3859215

[38] Centers for Disease Control Prevention. NHANES Survey Methods and Analytic Guidelines. Available online at: https://wwwn.cdc.gov/nchs/nhanes/analyticguidelines.aspx (accessed August 19, 2021).

[39] Lumley T. Analysis of complex survey samples. J Stat Softw. (2004) 9:1–19. 10.18637/jss.v009.i08

[40] Davis MA Murphy SP Neuhaus JM Lein D. Living arrangements and dietary quality of older US adults. J Am Diet Assoc. (1990) 90:1667–72. 10.1016/S0002-8223(21)01872-1 2246446

[41] Porter MP Stanford JL. Obesity and the risk of prostate cancer. Prostate. (2005) 62:316–21. 10.1002/pros.20121 15389806

[42] Centers for Disease Control Prevention. Specifying Weighting Parameters. Available online at: https://www.cdc.gov/nchs/tutorials/nhanes/surveydesign/weighting/intro.htm (accessed August 19, 2021).

[43] Crawford E Sutton SS Moul JW Pettaway CA Hardin JW Poston SA. Using prostate-specific antigen threshold to identify increased 4-year risk of prostate cancer. J Clin Oncol. (2010) 28:4565. 10.1200/jco.2010.28.15_suppl.456526753559

[44] Ross LE Taylor YJ Richardson LC Howard DL. Patterns in prostate-specific antigen test use and digital rectal examinations in the behavioral risk factor surveillance system, 2002-2006. J Natl Med Assoc. (2009) 101:316–24. 10.1016/S0027-9684(15)30878-6 19397221

[45] Giovannucci E. Tomato products, lycopene, prostate cancer: a review of the epidemiological literature. J Nutr. (2005) 135:2030S−1. 10.1093/jn/135.8.2030S 16046732

[46] Giovannucci E Clinton SK. Tomatoes, lycopene, and prostate cancer. Proc Soc Exp Biol Med. (1998) 218:129–39. 10.3181/00379727-218-44277 9605211

[47] Han GM Liu P. Higher serum lycopene is associated with reduced prevalence of hypertension in overweight or obese adults. Eur J Integr Med. (2017) 13:34–40. 10.1016/j.eujim.2017.07.002

[48] Beran TN Violato C. Structural equation modeling in medical research: a primer. BMC Res Notes. (2010) 3:267. 10.1186/1756-0500-3-267 20969789 PMC2987867

